# MiR395c Regulates Secondary Xylem Development Through Sulfate Metabolism in Poplar

**DOI:** 10.3389/fpls.2022.897376

**Published:** 2022-06-09

**Authors:** Chunhao Liu, Ding Ma, Zihao Wang, Ningcong Chen, Xiaoyun Ma, Xin-Qiang He

**Affiliations:** State Key Laboratory of Protein and Plant Gene Research, School of Life Sciences, Peking University, Beijing, China

**Keywords:** miR395, *ATPS*, sulfate metabolism, secondary xylem development, *Populus alba* × *P. glandulosa*

## Abstract

Secondary xylem development requires the coordination of multiple regulatory factors, including plant hormones, transcription factors, and microRNAs (miRNAs). MiR395 is an important regulator involved in sulfate metabolism, but its function in plant development is unclear. This study investigated the functions of miR395c in the secondary xylem development in *Populus alba* × *P. glandulosa*. MiR395c was highly expressed in the shoot apex and secondary xylem. The overexpression of miR395c resulted in an increase in both secondary xylem width and vessel dimension, as well as a decrease in the thickness of the secondary cell wall of the xylem fiber. Further analysis showed that miR395c inhibited biosynthesis of sulfate metabolic products by targeting *ATPS* genes, which led to the reduction of Abscisic acid (ABA) synthesis and down-regulation of *MYB46* expression. Our results indicate that miR395c regulates the secondary xylem development process via sulfate metabolism in *Populus*.

## Introduction

Secondary xylem, with components including tracheary elements, fibers, and parenchyma cells, transports water and inorganic salts and provides mechanical support for plant growth (Lucas et al., 2013). Xylem development is regulated by a variety of hormones and transcription factors. Auxin and cytokinin are key factors in the development of xylem; they participate in the xylem fate determination process by controlling the differentiation of meristematic stem cells (Bishopp et al., 2011; Gaillochet et al., 2017). Abscisic acid (ABA) is an important hormone regulating xylem development; it alters the morphology of xylem in *Arabidopsis* roots by activating miR165a, which inhibits the expression of *class III homeodomain leucine-zipper* (*HD-ZIP III*) transcription factors (Ramachandran et al., 2018). Recent studies have shown that ABA regulates xylem differentiation through the activation of different *vascular-related NAC-domain* (*VND*) transcription factors (Ramachandran et al., 2021). The R2R3 factor gene *MYB46* and *MYB83*, targeted and regulated by *VND6* and *VND7*, are the main transcription factors in the formation of secondary cell wall (SCW) (McCarthy et al., 2009; Kumar et al., 2016). *MYB46* and *MYB83* affect the formation of SCW by regulating the expression of downstream *MYB* transcription factors and other genes related to the SCW synthesis. Mutation of *MYB46* in *Betula platyphylla* leads to an increase in the dimension of xylem vessels and a decrease in the thickness of fiber secondary walls (Guo et al., 2017). It has been reported that ABA affects the formation of SCW by regulating *MYB* transcription factors through the activation of SNF1-RELATED PROTEIN KINASE 2 (SnRK2) to phosphorylate NAC SECONDARY WALL THICKENING PROMOTING FACTOR 1 (NST1) (Liu et al., 2021).

MicroRNAs (miRNAs), endogenous non-coding RNAs with a length of 20–24 nucleotide, are widely involved in many aspects of plant growth and development by specifically targeting complementary mRNA to downregulate gene expression (Curaba et al., 2014). Some miRNAs have been reported to play an important role in the formation of plant vascular tissues (Reinhart et al., 2002). MiR165 and miR166 affect shoot apical meristem (SAM) development and xylem differentiation by regulating the expression of *HD-ZIP III* transcription factors (Carlsbecker et al., 2010; Robischon, 2011; Zhu et al., 2011, 2013; Liu et al., 2015; Zhou et al., 2015). MiRNAs such as miR397, miR408, and miR857 affect SCW modification by regulating lignin biosynthesis (Wang et al., 2014; Zhao et al., 2015; Pan et al., 2018). However, the research on the role of miRNAs in the secondary vascular tissue development is still far from complete. MiR395c regulates the expression of three out of four members of the *ATP sulfurylase* (*ATPS*) gene family and the low affinity sulfate transporter *SULTR2;1* and plays an important role in the process of plant sulfur metabolism (Kawashima et al., 2009, 2011; Liang and Yu, 2010; Honsel et al., 2012; Matthewman et al., 2012; Ai et al., 2016; Yuan et al., 2016). *ATPS* gene family encodes enzymes catalyzing sulfate to adenosine 5′phosphosulfate (APS) in the first step of sulfate assimilation (Liang and Yu, 2010; Matthewman et al., 2012; Ai et al., 2016). APS is eventually transformed to cysteine in sulfate assimilation pathway (Matthewman et al., 2012; Batool et al., 2018). Sulfate and cysteine can promote ABA synthesis by acting as substrates of corresponding enzymes, which connects sulfate metabolism with the biosynthesis of ABA (Cao et al., 2014; Batool et al., 2018; Fatma et al., 2021). In addition, miR395c functions as an essential component in plant resistance to cadmium (Cd), *Verticillium dahliae* infection, and leaf spot disease (Zhang et al., 2013, 2017; Mu et al., 2018; Yang et al., 2020). However, miR395’s role in plant development remains unclear.

As secondary xylem represents the vast majority of woody biomass, it is of great importance to understand the principles behind secondary xylem development (Hou et al., 2020). In this study, we investigated the roles of miR395 in secondary xylem development of poplar.

## Materials and Methods

### Plant Materials and Transformation

Poplar 84K (*Populus alba* × *P. glandulosa*) was micropropagated on the Murashige and Skoog (MS) medium and cultivated in a glasshouse at 25°C under a 16 h:8 h, light:dark cycle, with 5,000 lux supplemental light and 60% relative humidity.

The latest databases of miRNAs (miRbase^1^) and poplar genome^2^ were used to retrieve sequence information for miRNAs and genes. Full-length cDNA of the *miR395c* precursor was obtained by gene cloning using gene-specific primers (Supplementary Table 1). The PCR products were cloned into the *pCAMBIA2300* vector by restriction enzyme digestion and enzyme linkage to generate *35S:miR395c*. Genetic transformation of poplar was performed using the leaf disk method mediated by *Agrobacterium* and selected using kanamycin (25 mg/L) (Jia et al., 2010).

### Material Collections and Quantitative Real-Time PCR

Poplars were micropropagated on MS for 2 months and transplanted to soil for 3 months post-cultivation, and then they were used for the phenotypic analysis.

Apex samples were collected under a stereomicroscope. Procambium and the vascular tissues at different developmental stages, including vascular cambium, secondary phloem, and secondary xylem, were isolated by tangential cryosectioning at –24°C with a Leica CM1850 Cryostat (Leica Microsystems Nussloch GmbH, Nussloch, Germany) (Zhang et al., 2011; Etchells et al., 2012). Three replicates of cryosections were collected for each developmental stage. The cryosections were frozen immediately in liquid nitrogen and stored at –70°C until RNA isolation. Total RNA was isolated with the RNeasy Plant Mini kit (Qiagen).

The internode above the first fully stretched leaf was defined as the first internode (Hou et al., 2020) and so on in this study. Half of each internode of transgenic poplar was fixed with formaldehyde alcohol acetic acid (FAA) solution (70% ethanol:glacial acetic acid:formaldehyde; 90:5:5, v/v) for the phenotype analysis, and the other half was quick-frozen in liquid nitrogen and stored in the –80°C refrigerator for the gene expression analysis. The leaves at 2nd, 4th, and 8th internodes were used to measure the water loss rate, and the remaining leaves were quick-frozen and stored.

Superreal Premix Plus (SYBR Green, QIAGEN) and MIRCute Plus miRNA qPCR (QIAGEN) kits were used to perform qPCR reaction to detect mRNA and miRNA levels in CFX96 Touch PCR (Bio-Rad). The amplification program consisted of 5 min of initial denaturation at 95°C, followed by 40 cycles of 10 s at 95°C, 32 s at 60°C, and ended with a final cooling step at 37°C for 30 s. Actin, UBQ, and U6 genes were used as internal references for mRNA and miRNA qPCR, respectively. The relative expression level was calculated using the delta-delta CT method (Livak and Schmittgen, 2001). qPCR primers are designed across exons to eliminate the effects of genomic DNA contamination (Supplementary Table 1).

### Water Loss and Stomatal Aperture Measurements

After determining the fresh weight (FW), the detached leaves were desiccated under normal atmospheric conditions. The desiccated weight of leaves was determined after exposure to air for 0.5, 1, 1.5, 2, 3, 5, and 8 h in a natural state, and the reduced weight (△W) within the corresponding time was obtained. The dry weight (DW) was determined after drying overnight in the oven at 80°C. Water loss rate (WLR, %) = [△W/(FW-DW)] × 100% (Guo et al., 2017).

Epidermal peels were stripped from the leaves of 3-month soil-grown *miR395c*-OE and WT plants and observed under a microscope. The width and length of each observable stoma were measured by ImageJ^3^. The ratios of the widths and lengths of stomatal apertures under different treatments were calculated (Guo et al., 2017).

### Histological Analysis

After freehand sectioning of poplar internodes, the stem sections were stained with 0.1% (w/v) toluidine blue O (Sigma) to examine the xylem phenotype by using light microscopy (Mitra and Loque, 2014). Lignin distribution was observed by phloroglucinol-HCl staining (Mitra and Loque, 2014). The nitric acid-chromic acid separation method was used to measure the length and width of xylem cells (Hamann et al., 2011).

Confocal laser microscope (Olympus) was used to observe lignin autofluorescence. The excitation light wavelength is 405 nm, and the receiving light range is 430–470 nm. Bright field was observed under a Zeiss Axioskop 2 Plus microscope equipped with a computer-assisted digital camera. The 0.2-mm-thick internode sections of 3-month-old *miR395c*-OE and WT 84K poplar plants were obtained manually for scanning electron microscopy (SEM, Quanta FEG 450, FEI, United States).

The vessel dimension and the width and length of separated cells from 10 anatomical sections were measured using the ImageJ software. Fiber SCW thickness was measured under SEM using its own micrometer. Each micro-measurement value in this article was an average of more than 30 original data after deleting extreme values.

### Determination of Cell Wall Composition

The lignin, cellulose, and hemicellulose levels of 3-month-old *miR395c*-OE and WT poplar stems were determined by using enzyme-linked immunosorbent assay (ELISA) kits (lignin ELISA kits, MM-0886O2; cellulose ELISA kits, MM-36065O2; hemicellulose ELISA kits, MM-37041O2; Meimian Biotechnology Co., Ltd., China).

### Determination of Gibberellin and Abscisic Acid Contents

The contents of gibberellin and ABA were determined by using the ELISA method (Wang et al., 2021). Plant tissues (i.e., stems and leaves) were fully ground in liquid nitrogen and then added with PBS buffer. The supernatant of the mixture was taken for ELISA experiments, and the absorbance of treated solution was measured using a NanoPhotometer.

### Treatment With Sulfur or Abscisic Acid in Poplar Seedlings

MiR395c-OE and wild-type 84K poplar seedlings were cultivated at different growth conditions, including sulfur deficiency and sulfur or ABA surplus. Sulfur-deficient culture, normal culture, and sulfur-added culture contain 750 μM, 1.5 mM, and 3 mM sulfate, respectively. The ABA-treated medium contains 2 μM ABA. MiR395c-OE and wild-type 84K poplars were micropropagated on the above different culture medium for 2 months, and then they were used for phenotypic analysis.

## Results

### Expression Pattern of MiR395c and Its Target *ATPS* Genes During Vascular Development in Poplar

**FIGURE 1:**
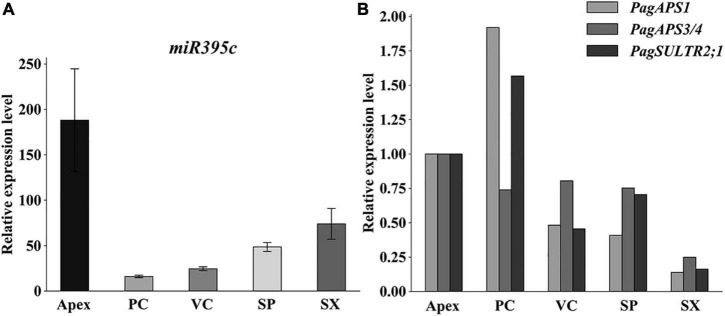
Expression pattern of miR395c, *PagATPS*, and *PagSULTR2;1* during vascular development in poplar. **(A)** qPCR analysis showing that the expression of mature miR395c in the apex and secondary xylem was higher than in the procambium, vascular cambium, and secondary phloem. **(B)** qPCR analysis showing that the expression of *PagAPS1*, *PagAPS3/4*, and *PagSULTR2;1* in the cambium and secondary phloem was higher than in the secondary xylem. *U6* and *UBQ* or *actin* was used as internal reference genes in miRNA qPCR and mRNA qPCR analysis, respectively. Error bars represent the standard error of three independent biological replicates. PC, procambium; VC, vascular cambium; SP, secondary phloem; SX, secondary xylem.

The miR395 family in *P. trichocarpa* has 11 members, all of which have a 21 base-pair (bp) mature sequence (miRbase: see text footnote 1; Supplementary Figure 1). As predicted by the RNAfold Web server (Gruber et al., 2008), precursors of the poplar miR395 members form stem-loop secondary structures (Supplementary Figure 1) and generate a similar mature miRNA (Supplementary Figure 1). MiR395 is grouped into two categories with respect to their mature sequences. MiR395b-k share the same mature sequence, but they differ from miR395a in two bases. Based on the poplar RNAseq analysis of apex, procambium, cambium, secondary phloem, and secondary xylem isolated by tangential cryosectioning, it was found that the expression level of miR395c in the apex and secondary xylem was higher than that in the procambium, cambium, and secondary phloem (Figure 1A). By multiple sequence alignment of *AtAPS1*, *AtAPS3*, *AtAPS4*, and *AtSULTR2;1* which were the target genes of miR395c in *Arabidopsis*, homologous proteins in poplar were identified. Potri.008G159000.3 (*PagAPS1*), Potri.010G081200.1 (*PagAPS3/4*), and Potri.002G092400.1 (*PagSULTR2;1*) were mapped to *AtAPS1*, *AtAPS3*/*AtAPS4*, and *AtSULTR2;1*, respectively, and had the target sites of miR395c (Supplementary Figure 2). During the development of the vascular tissue in 84K poplar, *PagAPS1* and *PagAPS3/4* had a higher expression in the procambium and apex. Meanwhile, their expression in the cambium and secondary phloem was higher than the secondary xylem (Figure 1B). The expression level of *PagSULTR2;1* in secondary xylem was also lower than in the vascular cambium and secondary phloem (Figure 1B). The expression patterns of miR395c target genes *PagAPS1*, *PagAPS3/4*, and *PagSULTR2;1* in vascular tissues were exactly opposite to miR395c.

### MiR395c Regulates Secondary Xylem Development

**FIGURE 2:**
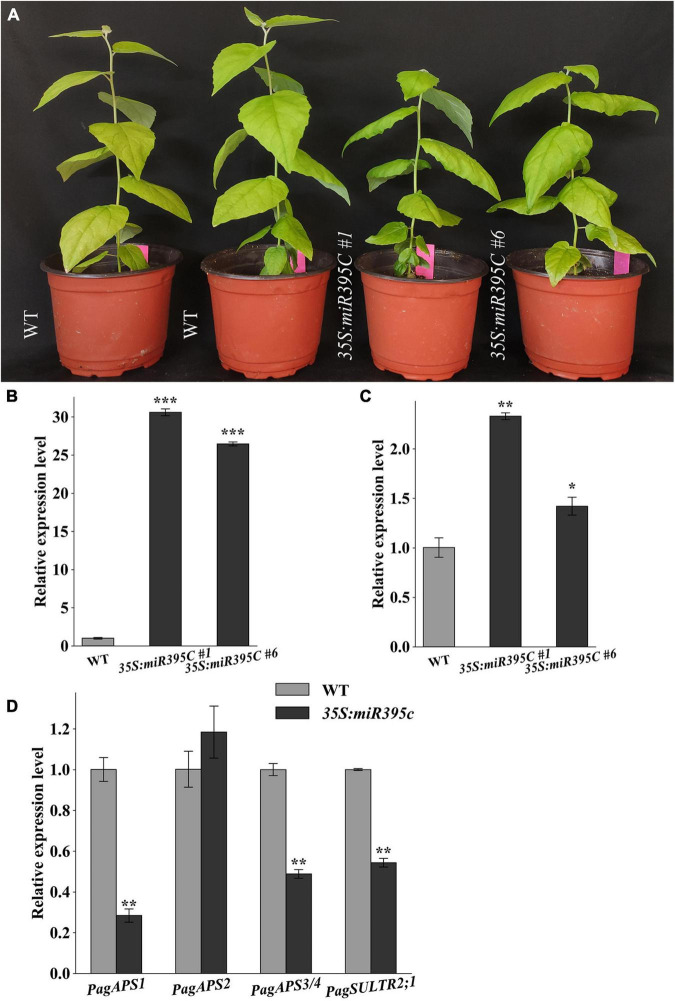
Expression analysis of miR395c and its targets in miR395c-OE poplars. **(A)** The height of transgenic plants and wild type. **(B)** The expression of precursor miR395c increased in miR395c-OE poplars. **(C)** The expression of mature miR395c increased in miR395c-OE poplars. **(D)** The expression of *PagAPS1*, *PagAPS3/4*, and *PagSULTR2;1* decreased in the transgenic plants, while the expression of *PagAPS2* changed slightly. Asterisks indicate significant difference between transgenic and WT plants using the Student’s *t*-test, *p < 0.05; **p < 0.01; and ***p < 0.001.

To investigate the function of miR395c in vascular development, *35S:miR395c* 84K poplar lines were generated. A total of 8 miR395c-overexpressing (OE) lines were obtained, two of which (i.e., OE#1 and OE#6) were selected for further analysis (Figure 2A). Compared with wild type (WT), the height of *35S:miR395c* 84K poplar decreased significantly (Figure 2A). qPCR analysis showed that the expression of precursor and mature miR395c increased in miR395c-OE lines (Figures 2B,C), while the expression of miR395c target genes *PagAPS1*, *PagAPS3/4*, and *PagSULTR2;1* decreased (Figure 2D).

**FIGURE 3:**
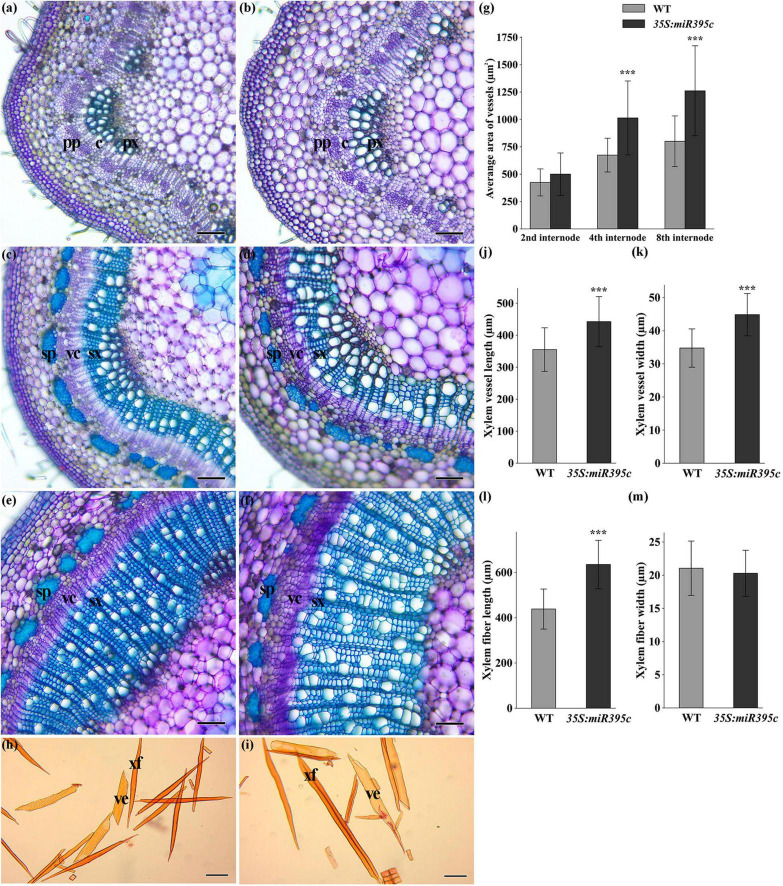
Cross-sections of 2nd, 4th, and 8th internodes in 3-month-old wild-type and transgenic 84K poplars. **(a)** Cross-section of the 2nd internode in wild type. **(b)** Cross-section of the 2nd internode in miR395c-OE poplar. **(c)** Cross-section of the 4th internode in wild type. **(d)** Cross-section of the 4th internode in miR395c-OE poplar. **(e)** Cross-section of the 8th internode in the wild type. **(f)** Cross-section of the 8th internode in miR395c-OE poplar. pp, primary phloem; px, primary xylem; c, cambium; vc, vascular cambium; sp, secondary phloem; sx, secondary xylem. **(g)** Average area of vessels increased in transgenic plants. **(h)** Separation and safranin staining of secondary xylem cells in wild-type poplar. **(i)** Separation and safranin staining of secondary xylem cells in miR395c-OE poplar. Bar, 100 μm. xf, xylem fiber; ve, vessel. **(j)** Xylem vessel length increased in miR395c-OE poplar. **(k)** Xylem vessel width increased in miR395c-OE poplar. **(l)** Xylem fiber length increased in miR395c-OE poplar. **(m)** Xylem fiber width changed slightly in miR395c-OE poplar. Asterisks indicate significant difference between transgenic and WT plants using the Student’s *t*-test, ***p < 0.001.

**FIGURE 4:**
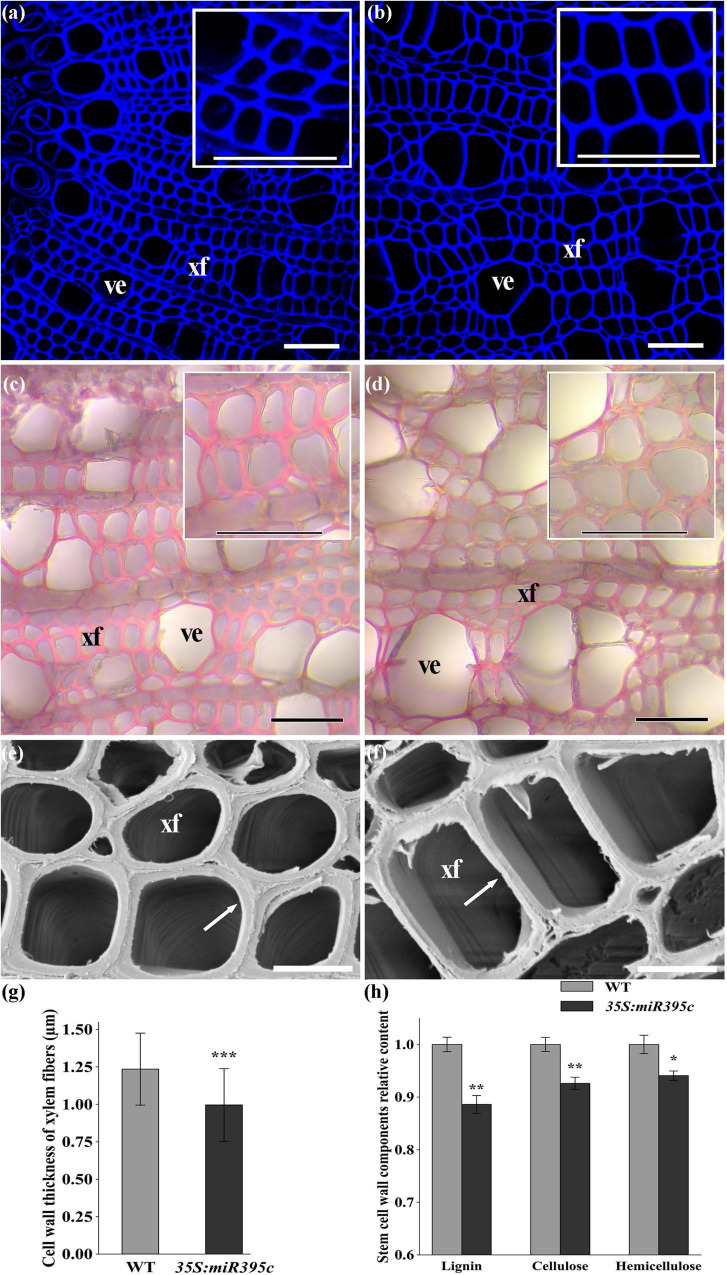
The SCW of the 8th internode of 3-month-old wild type and transgenic poplar. **(a)** Lignin autofluorescence of wild type. **(b)** Lignin autofluorescence of miR395c-OE poplar. Phloroglucinol-HCL staining of **(c)** wild-type and **(d)** miR395c-OE poplar. Bar, 50 μm. **(e)** Electron microscopy on the xylem in wild type. **(f)** Electron microscopy on the xylem in miR395c-OE poplar. The arrow refers to the wall thickness of the xylem fiber. Bar, 10 μm. xf, xylem fiber; ve, vessel. **(g)** The thickness of fiber cell wall decreased in miR395c-OE poplar. **(h)** The content of lignin, cellulose, and hemicellulose decreased in miR395c-OE poplar. Insets in **(a–d)** are high-magnification images of the middle xylem area. Asterisks indicate significant difference between transgenic and WT plants using the Student’s *t*-test, *p < 0.05; **p < 0.01; ***p < 0.001.

Microscopic observation revealed that *35S:miR395c* 84K poplar displayed distinct phenotypes in terms of xylem development. Notably, in 3-month-old *35S:miR395c* 84K poplar, the xylem width and vessel dimension increased significantly at 2nd, 4th, and 8th internodes compared with WT (Figures 3a–f). The statistical analysis of vessel average area found that the vessel average area of 2nd, 4th, and 8th internodes in miR395c overexpression plants increased by 17.7, 50.6, and 57.7%, respectively (Figure 3g), among which the vessel average area of 4th and 8th internodes increased significantly. By nitric acid-chromic acid separation and safranin staining of the xylem, individual vessels, xylem fibers, and ray cells can be observed clearly (Figures 3h,i). The length and width of the vessels and the length of fibers in miR395c-OE 84K poplar increased by 24.6, 29.0, and 44.9%, respectively, but the fiber width had a slight change (Figures 3j–m). In addition, autofluorescence and electron microscopy showed that the overexpression of miR395c resulted in the reduction of xylem fiber cell wall thickness (Figures 4a,b,e,f). The thickness of *35S:miR395c* 84K poplar fiber cell wall was reduced by 19.4% on average compared with that of WT (Figure 4g). After phloroglucinol-HCl staining, xylem of *35S:miR395c* 84K poplar displayed a less intense red coloration than WT (Figures 4c,d). This result indicated that the overexpression of miR395c led to a decrease in lignin deposition. Further analysis of secondary wall components showed that the content of lignin, cellulose, and hemicellulose dropped dramatically in miR395c-OE 84K poplar (Figure 4h).

### MiR395c Affects Abscisic Acid Synthesis and the Expression of *MYB46* Through Sulfur Metabolism Pathway in Secondary Xylem Development

**FIGURE 5:**
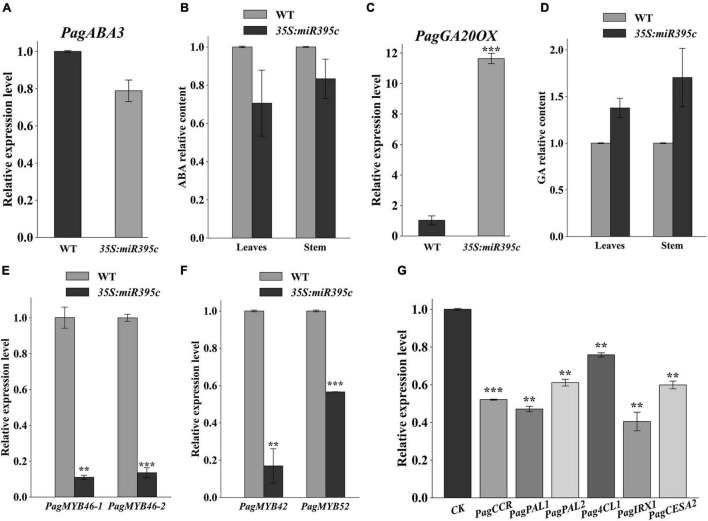
The content of abscisic acid (ABA) and gibberellic acid (GA) in wild type and miR395c-OE poplars, and the expression of genes involved in secondary wall deposition. **(A)** Relative expression of *PagABA3* related to ABA biosynthesis in miR395c-OE poplar. **(B)** The content of ABA decreased in miR395c-OE poplar. **(C)** Relative expression of *PagGA20OX* related to GA biosynthesis in miR395c-OE poplar. **(D)** The content of GA increased in miR395c-OE poplar. **(E)** The expression level of *PagMYB46* in miR395c-OE poplar. **(F)** The expression levels of *PagMYB42* and *PagMYB52* in miR395c-OE poplar. **(G)** Relative expression of genes involved in secondary wall biosynthesis in miR395c-OE poplar. CK, control check. Asterisks indicate significant difference between transgenic and WT plants using the Student’s *t*-test, **p < 0.01; ***p < 0.001.

In miR395c-OE 84K poplar, the water loss rate of leaves at 4th and 8th nodes was higher (Supplementary Figure 3), and the stomatal aperture was larger than WT (Supplementary Figures 3a–c). Since ABA is an important factor regulating the opening and closing of stoma, the result above indicated that miR395c may affect the ABA synthesis. The expression of *ABA3*, a gene involved in the ABA synthesis, decreased in miR395c-OE poplar (Figure 5A), and the ABA level was also lower in both stems and leaves (Figure 5B). As previously described, miR395c-targeted genes *ATPS* were involved in the regulation of the ABA biosynthesis through controlling sulfate assimilation, suggesting a high probability that miR395c inhibits ABA synthesis by limiting sulfate assimilation. Gibberellin [gibberellic acid (GA)] is another important hormone that promotes cell elongation and expansion, reported to have an antagonistic relationship with ABA. This study found that the overexpression of miR395c resulted in a significant increase in the length and width of vessels, as well as the length of xylem fibers (Figures 3k–m). MiR395c may also affect the synthesis of GA. The overexpression of miR395c upregulated the expression of *GA20OX* and increased the content of GA in stems and leaves (Figures 5C,D).

A previous study had shown that the mutation of *BplMYB46* in *B. platyphylla* led to an increase in the dimension of xylem vessels and a decrease in the thickness of fiber secondary walls and affected the rate of leaf water loss and stomatal aperture (Guo et al., 2017), which was similar to the results we had obtained in this article. To investigate whether *MYB46* participated in the formation of the phenotypes of miR395c-OE 84K poplar, we did a qPCR analysis and found that the expression level of *PagMYB46-1* and *PagMYB46-2* in transgenic lines were decreased as predicted (Figure 5E).

In addition, we also examined the expression of *MYB46* downstream transcription factors and their regulated genes related to SCW biosynthesis. *MYB42* and *MYB52*, which are the downstream transcription factors of *MYB46*, directly activate lignin and phenylalanine biosynthesis genes during the SCW formation. The expression of *PagMYB42* and *PagMYB52* decreased in the miR395c-OE poplar (Figure 5F). *Cinnamoyl-CoA reductase* (*CCR*), *phenylalanine ammonia lyase* (*PAL*), *4-coumarate-coa ligase* (*4CL*), *irregular xylem* (*IRX*), and *cellulose synthase* (*CESA*) are homologous to lignin, hemicellulose, and cellulose biosynthesis genes. The expression of those genes targeted and regulated by *MYB42* and *MYB52* decreased in miR395c-OE poplar, which agrees with the reduced secondary wall synthesis (Figure 5G).

### Sulfur and Abscisic Acid Complement the Secondary Xylem Phenotype of MiR395c-OE

**FIGURE 6:**
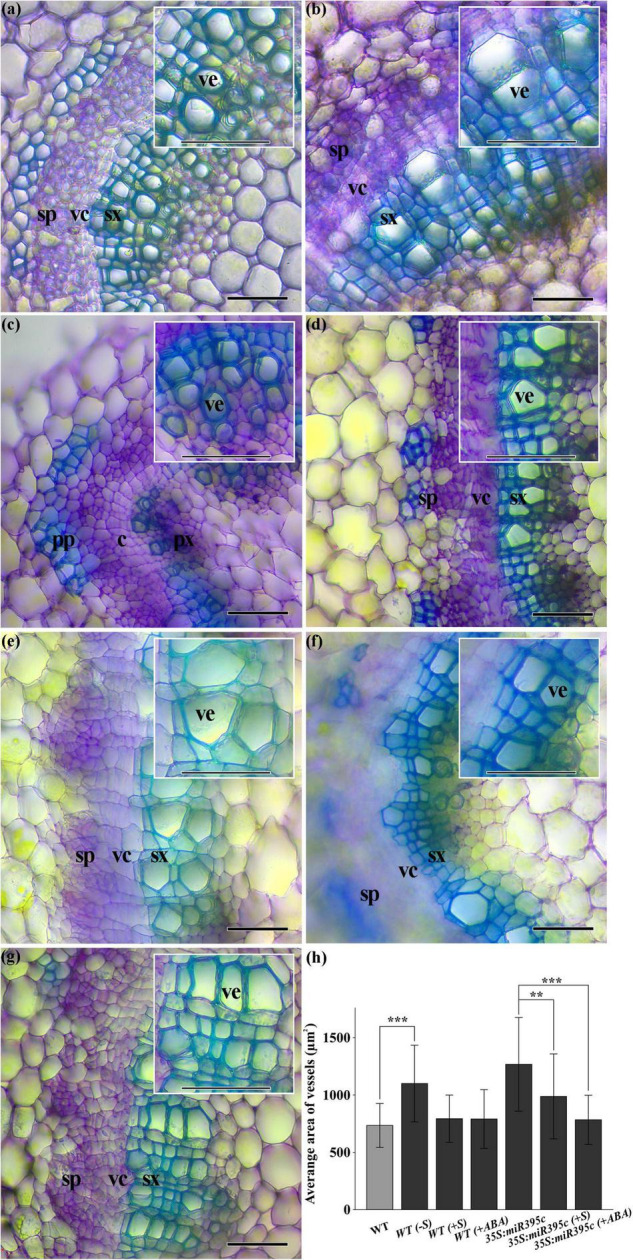
Cross-sections of the 6th internode of 2-month-old wild-type and miR395c-OE 84K poplars treated with sulfur deficiency and sulfur or ABA surplus. **(a)** Cross-section of the wild type. **(b)** Cross-section of the wild type treated with sulfur deficiency. **(c)** Cross-section of the wild type added with sulfur. **(d)** Cross-section of the wild type added with ABA. **(e)** Cross-section of the miR395c-OE poplar. **(f)** Cross-section of the miR395c-OE poplar added with sulfur. **(g)** Cross-section of the miR395c-OE 84K poplar added with ABA. pp, primary phloem; px, primary xylem; c, cambium; vc, vascular cambium; sp, secondary phloem; sx, secondary xylem; ve, vessel. **(h)** Vessel dimension increased in wild type plants treated with sulfur deficiency and decreased in miR395c-OE plants added with sulfur or ABA. Bar, 50 μm. Asterisks indicate significant difference between transgenic and WT plants using the Student’s *t*-test, **p < 0.01; ***p < 0.001.

To confirm the role of sulfur and ABA in the development of the poplar secondary xylem, miR395c-OE and WT 84K poplar seedlings were cultivated at different growth conditions including sulfur deficiency, sulfur, or ABA surplus, respectively. The dimension of xylem vessels was significantly increased, and the thickness of xylem fiber cell wall was decreased in the 2-month-old WT 84K poplar under sulfur deficiency conditions (Figures 6a,b). The xylem phenotype of 84K poplar with sulfur deficiency was similar to miR395c-OE 84K poplar. The dimension of vessels did not change significantly after cultivated with sulfur or ABA, but the cell wall thickness was still greater than WT under sulfur deficiency conditions and miR395c-OE 84K poplar (Figures 6a,c,d). Compared with normally cultured miR395c-OE plants, the dimension of vessels was significantly reduced, and the thickness of fiber cell wall increased in the miR395c-OE poplar with external supply of sulfur or ABA (Figures 6e–g). In addition, sulfur deficiency in WT or miR395 overexpression repressed the expression of *ATPS* and SCW biosynthesis-related genes, whereas sulfur surplus in WT or miR395c-OE poplars induced the expression of these genes (Supplementary Figure 4). These results indicated that 84K poplar under sulfur deficiency conditions exhibited a similar xylem phenotype with miR395c-OE plants. Meanwhile, external supply of sulfur or ABA partially complemented the xylem phenotype of miR395c-OE poplar (Figure 6h). The results above confirmed that miR395c regulates the development of secondary xylem exactly through sulfate metabolism and ABA pathway in 84K poplar.

## Discussion

As the product of the secondary xylem development, wood has become one of the most important sustainable energy sources. The development of secondary xylem in woody plants requires the participation of various regulatory factors, including hormones, transcription factors, and miRNAs (Robischon, 2011).

MiR395c regulates the process of plant sulfur metabolism by its targets *ATPS* genes (Liang and Yu, 2010; Kawashima et al., 2011; Matthewman et al., 2012; Ai et al., 2016; Yuan et al., 2016). Overexpression of miR395c in Arabidopsis inhibits the activation of sulfate assimilation through negative regulation of *APS1*, *APS3*, and *APS4* genes and disrupts the sulfate transport from mature leaves to young leaves through negative regulation of *SULTR2;1*, while the expression of *APS2* is unaffected (Liang and Yu, 2010; Matthewman et al., 2012; Ai et al., 2016).

Sulfur is one of the six macronutrients necessary for plant growth and participates in many important physiological and biochemical processes (Matthewman et al., 2012; Ai et al., 2016; Yuan et al., 2016). Sulfate enters the cell to form APS, which is then used in the synthesis of cysteine (Matthewman et al., 2012; Batool et al., 2018). Cysteine is an essential amino acid in plants. Cysteine promotes the biosynthesis of ABA by regulating the expression of *NINE*-*CIS*-*EPOXYCAROTENOID DIOXYGENASE 3* (*NCED3*) and *ABA3* (Cao et al., 2014; Batool et al., 2018; Fatma et al., 2021). In the process of sulfate assimilation, *ATPS*, targeted by miR395c, is responsible for the activation of sulfate to form APS. In miR395c-OE 84K poplars, the expression of *PagAPS1* and *PagAPS3/4* decreased, and sulfate assimilation was restricted (Figure 2D). In addition, the ABA content in stems and leaves of miR395c-OE plants decreased, the stomatal aperture of the leaves increased, and the water loss rate increased (Supplementary Figures 3a–d). Recent studies have found that ABA activates *SnRK2* by binding to the PYR/PYL receptor and the type 2C protein phosphatase 2C (PP2C) complex, which promote the expression of *MYB46* by phosphorylating NST1 (Liu et al., 2021). *MYB46* is the main transcription factor that regulates SCW formation and lignin deposition (McCarthy et al., 2009). The expression levels of *PagMYB46-1* and *PagMYB46-2* in miR395c-OE 84K poplars decreased (Figure 5E). Interestingly, the mutation of *B. platyphylla BplMYB46* resulted in an increase in the dimension of vessels and a decrease in the thickness of xylem fiber SCW, as well as an increase in both leaf water loss rate and stomatal aperture (Guo et al., 2017), which showed a similar phenotype to miR395c-OE 84K poplar.

**FIGURE 7:**
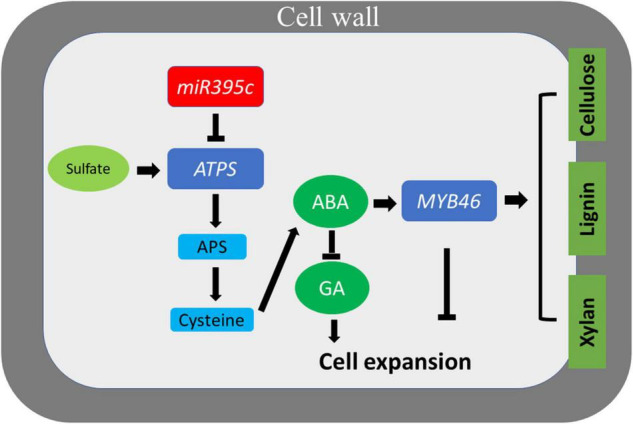
A model for the role of miR395c in the development of xylem. Overexpression of miR395c downregulates *ATPS*, which then inhibits sulfate assimilation and synthesis of sulfate metabolic product cysteine. The decrease in cysteine inhibits the synthesis of ABA, which leads to an increase in the dimension of xylem vessels and a decrease in the thickness of fiber secondary walls by suppressing the expression of *MYB46*. Increased GA, which is antagonistic to ABA, promotes cell expansion in miR395c-OE poplar.

In this study, we proposed a model that MiR395c regulates the biosynthesis of ABA through the sulfate metabolism pathway, which subsequently affects the expression of MYB46 and regulates the secondary xylem development (Figure 7). MiR395c inhibits the synthesis of sulfate metabolic product cysteine by negatively regulating the expression of *ATPS*. The decrease in the cysteine synthesis inhibits the synthesis of ABA, reduces the thickness of xylem fiber SCW, and increases the dimension of vessels by suppressing the expression of *MYB46*. In addition, the content of GA, which is antagonistic to ABA, promotes cell elongation, and expansion, increased in miR395c-OE plants. WT 84K poplar under sulfur deficiency conditions showed a similar xylem phenotype with miR395c-OE plants and the xylem phenotype of miR395c transgenic 84K poplar was partially complemented after adding sulfur or ABA, which further verified our conclusions.

In conclusion, this study found that miR395c regulates the secondary xylem development by affecting the biosynthesis of ABA and the expression of *MYB46* through regulating sulfate assimilation, which provides the foundation for future research on the relationship between sulfate metabolism and vascular tissue development.

## Data Availability Statement

The original contributions presented in the study are included in the article/Supplementary Material, further inquiries can be directed to the corresponding author/s.

## Author Contributions

CL and DM performed the experiments and assisted with data analyses. X-QH designed the experiments, analyzed, and interpreted the data. NC, ZW, and XM assisted with data analyses. CL, DM, and X-QH wrote the manuscript. All authors read and commented on the manuscript.

## Conflict of Interest

The authors declare that the research was conducted in the absence of any commercial or financial relationships that could be construed as a potential conflict of interest.

## Publisher’s Note

All claims expressed in this article are solely those of the authors and do not necessarily represent those of their affiliated organizations, or those of the publisher, the editors and the reviewers. Any product that may be evaluated in this article, or claim that may be made by its manufacturer, is not guaranteed or endorsed by the publisher.

## Footnotes

1
http://www.mirbase.org

2
https://phytozome-next.jgi.doe.gov

3
http://rsbweb.nih.gov/ij/
