# Supplementary material for: MiR395c Regulates Secondary Xylem Development Through Sulfate Metabolism in Poplar
Source: Front Plant Sci. 2022 Jun 9;13:897376. doi: 10.3389/fpls.2022.897376 (PMC9218717; doi:10.3389/fpls.2022.897376)
Supplement: Supplementary file 2 [file Data_Sheet_1.docx]

Supporting Information


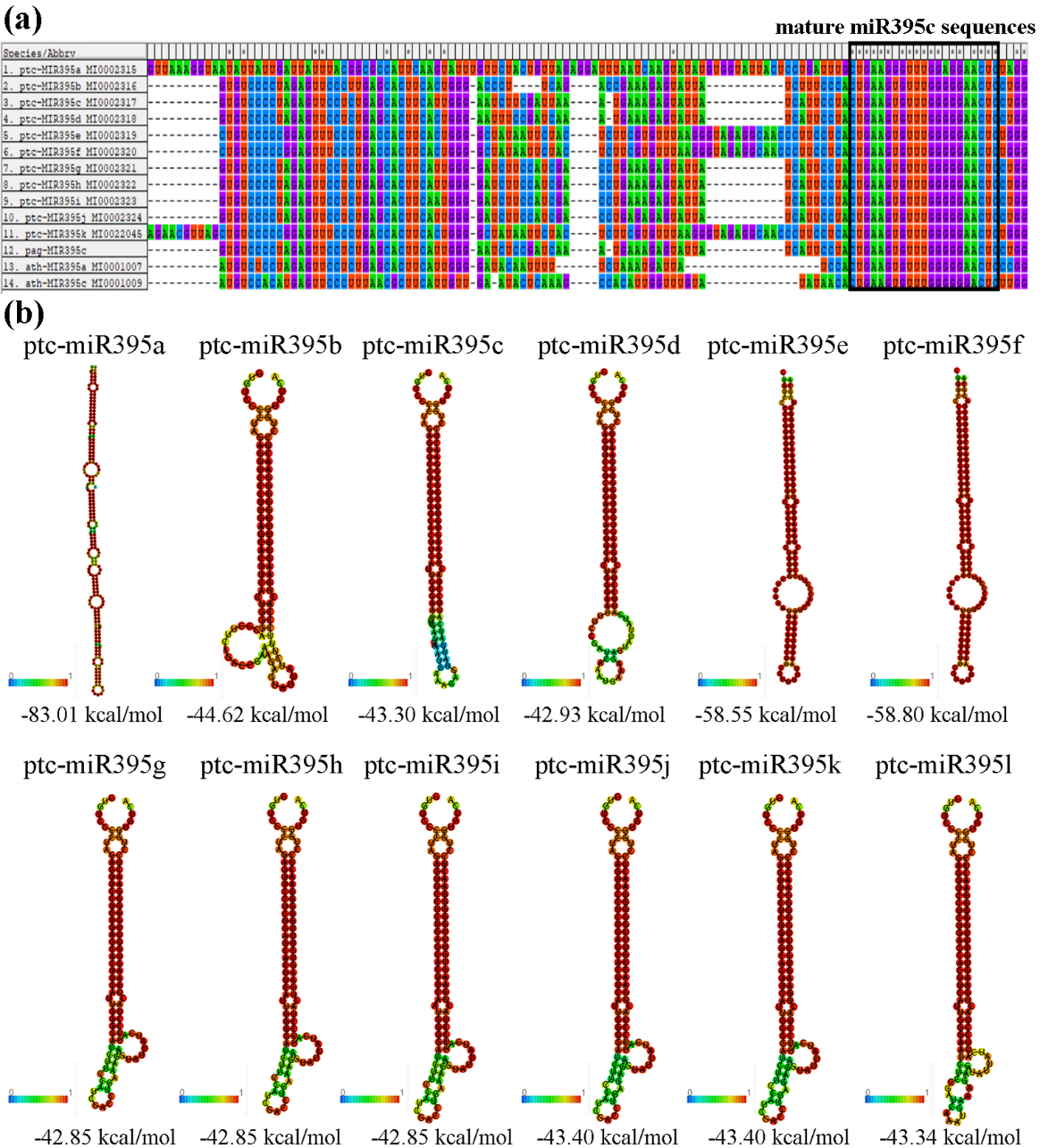


**Fig. S1 Sequence analysis and secondary structure prediction of miR395 family in *Populus.***

(a) Sequence alignment of stem-loop precursors of miR395 members from *Populus* and *Arabidopsis*. (b) Predicted stem-loop secondary structures of miR395 members in poplar. Stem-loop secondary structures were predicted with the RNAfold WebServer (Gruber *et al*., 2008) using default parameters. The structures were colored by base-pairing probabilities. The minimum free energy of each secondary structure and the nucleotide sequence of the precursors of poplar miR395 were indicated.


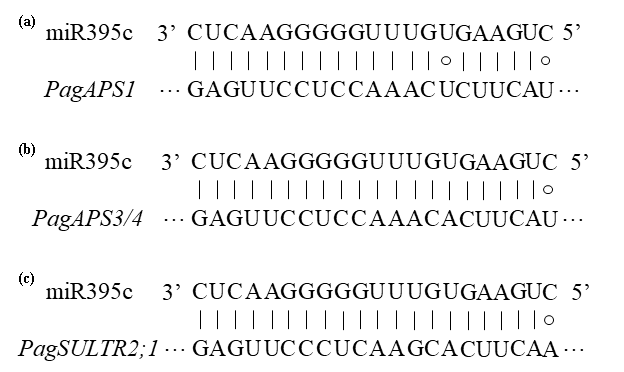


**Fig. S2 Target sites of the three miR395c target genes in poplar.**

(a) Sequence alignment of miR395c and its target gene *PagAPS1*. (b) Sequence alignment of miR395c and its target gene *PagAPS3/4*. (c) Sequence alignment of miR395c and its target gene *PagSULTR2;1*.


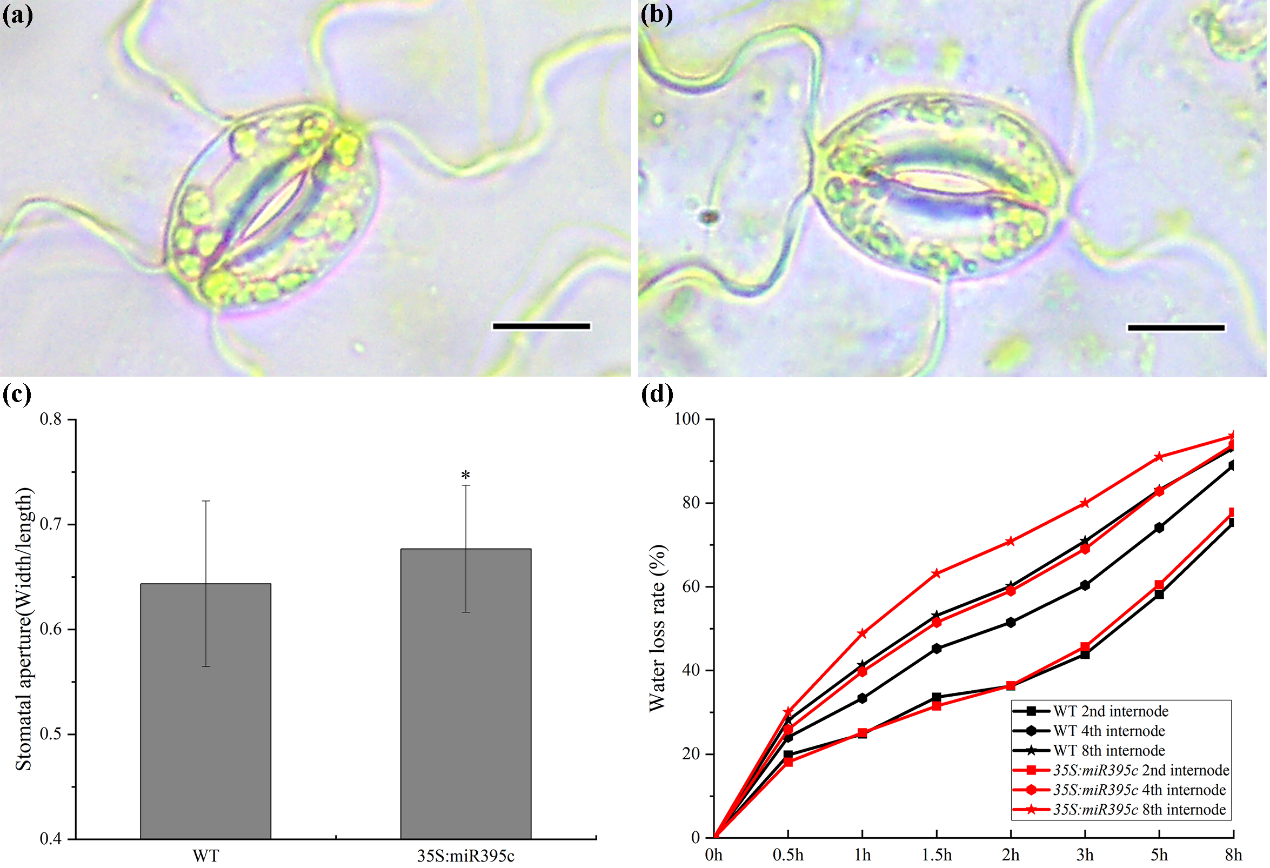


**Fig. S3 Stomata aperture and water loss rate of leaves in wild type and miR395c-OE poplar.**

(a) The stoma of wild type poplar. (b) The stoma of overexpression poplar. Bar, 10µm. (c) The stomata aperture increased in the *35S:miR395c* 84K poplar. (d) The water loss rate of leaves increased at 4^th^ and 8^th^ nodes of miR395c-OE poplar.


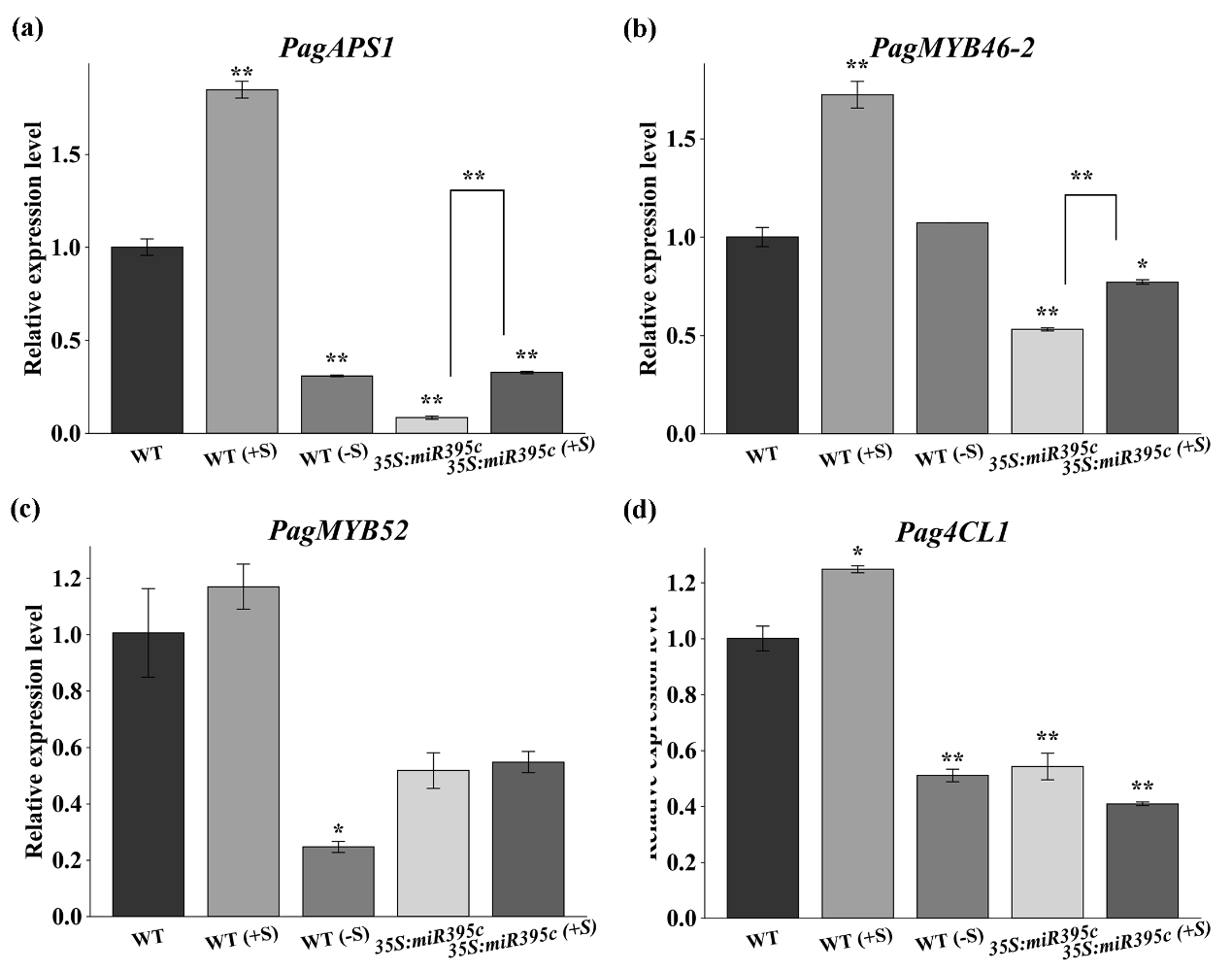


**Fig.** **S4 Expression levels of *ATPS* and SCW biosynthesis-related genes in wild type and miR395c-OE poplars under sulfur deficiency or sulfur surplus condition.**

(a) Relative expression of *PagAPS1* in wild type and miR395c-OE poplars under sulfur deficiency or sulfur surplus condition. (b) Relative expression of *PagMYB46-2* in wild type and miR395c-OE poplars under sulfur deficiency or sulfur surplus condition. (c) Relative expression of *PagMYB52* in wild type and miR395c-OE poplars under sulfur deficiency or sulfur surplus condition. (d) Relative expression of *Pag4CL1* in wild type and miR395c-OE poplars under sulfur deficiency or sulfur surplus condition. Asterisks indicate significant difference between transgenic and WT plants using Student’s t-test. *, p<0.05; **, p<0.01.
